# Changes in the ocular surface and meibomian glands following administration of 0.01 and 0.02% atropine

**DOI:** 10.3389/fmed.2025.1585396

**Published:** 2025-06-24

**Authors:** Xueyan Li, Yanying Zhu, Haiyan Xie, Yuexin Chen, Yukun Liu, Xiaochen Xu, Jing Wang

**Affiliations:** ^1^The Second Affiliated Hospital of Anhui Medical University, Anhui Medical University, Hefei, China; ^2^Wuhu City Eye Hospital, Wuhu, China

**Keywords:** low-concentration atropine, myopia, children, ocular surface, meibomian gland bio-image analyzer

## Abstract

**Purpose:**

To investigate the changes in the ocular surface and meibomian glands following the administration of 0.01 and 0.02% atropine.

**Setting:**

The Second Affiliated Hospital of Anhui Medical University, Anhui, China.

**Methods:**

In this randomized controlled study, the 0.01% group (18 patients, 36 eyes) and the 0.02% group (15 patients, 30 eyes) underwent assessments using the Ocular Surface Disease Index (OSDI), Visual Analogue scale (VAS), tear meniscus height (TMH), first noninvasive tear film breakup time (fNIBUT), and average noninvasive tear film breakup time (avNIBUT) at baseline (before administration) and at 1, 3, 6, and 12 months post-treatment. The meibomian glands of both the upper (U) and lower (L) eyelids were evaluated using Meibomian Gland Bio-image Analyzer, measuring parameters such as average gland diameter (avGD), average gland length (avGL), average gland area (avGA), deformation coefficient (DC), and gland visibility score (VS).

**Results:**

There were no statistically significant differences in any of the parameters within the 0.01% group (all *p* > 0.05). In the within-group comparisons of the 0.02% group, OSDI was higher at 3 month (*p* = 0.006) and was lower at 12 months (*p* = 0.038). VAS was higher at 3 months and 6 months (all *p* < 0.05). TMH was lower at 12 months (all *p* < 0.05). U-VS was lower at 3 months (*p* = 0.006) and higher at 6 and 12 months (all p < 0.05). L-avGL was higher at 1 month (*p* = 0.001) and lower at 6 months (*p* = 0.013). L-VS was higher at 6 and 12 months (all *p* < 0.05). In the 0.02% group, at 3 months, the change in U-VS and L-VS were positively correlated with the change in the VAS (r = 0.542, *p* = 0.037; r = 0.614, *p* = 0.015). At 6 months, the change in L-VS was positively correlated with the change in OSDI (r = 0.610, *p* = 0.016). At 12 months, the change in U-VS was positively correlated with the change in TMH (r = 0.521, *p* = 0.003).

**Conclusion:**

0.01% atropine had no significant impact. 0.02% atropine eye drops affected the lipid secretion of meibomian glands, tear meniscus height and subjective discomfort.

## Introduction

The widespread use of electronic devices and increasing educational pressure have made the prevention and control of myopia a global public health challenge that demands immediate attention. The proportion of adolescents within the myopic population has been increasing year by year (1, 2). From 1990 to 2023, the prevalence of myopia in children has been higher than that in adolescents. It is projected that by 2050, the global prevalence of myopia will increase to 39.8%, affecting more than 740 million children and adolescents (3). At the same time, high myopia is often classified as pathological myopia, which is prone to complications such as posterior scleral staphyloma, macular degeneration, choroidal neovascularization, and a series of blinding eye diseases (4). Currently, low-concentration atropine eye drops are commonly used to manage myopia progression and axial elongation (5).

Clinical studies (6–10) have demonstrated that low-concentration atropine eye drops can significantly retard the progression of myopia, with relatively minimal side effects and a higher degree of patient compliance. Compared to high-concentration atropine, the side effects of low-concentration atropine, such as difficulty with near accommodation and glare, are less severe, resulting in better patient acceptance. Therefore, low-concentration atropine ophthalmic solution is considered a relatively safe and effective approach for the prevention and management of myopia, especially in adolescents.

Dry eye disease is an ocular surface disorder usually caused by various factors. Its main manifestation involves irregularities in the quality, secretion, and dynamics of tear fluid, leading to tear film instability and disrupting the ocular surface microenvironment. This condition may also be linked to ocular surface inflammation, tissue damage, and neurofunctional disturbances, resulting in discomfort or impairing visual function. Currently, there is no definitive conclusion regarding the long-term use of atropine in children and its potential effects on the meibomian glands and tear film, which could contribute to dry eye disease. Animal studies have shown that 1% atropine eye drops can quickly induce dry eye symptoms in rabbit eyes, although this effect tends to diminish after several weeks (11). Li M et al. (12) reported that in a population of myopic adults in China, short-term use of low-concentration atropine eye drops initially led to a significant reduction in tear meniscus height and an increase in dry eye scores. However, symptoms were alleviated 18 h after use. Atropine may inhibit lacrimal gland secretion. After using 0.01% atropine eye drops, both the tear secretion test results and the tear film break-up time showed a decreasing trend (13).

With the increasing use of low-concentration atropine eye drops to control myopia progression, the potential link between atropine and dry eye disease has gained attention. This study seeks to investigate the effects of different concentrations of atropine eye drops on ocular surface health and meibomian gland function in children.

## Patients and methods

### Patients

**Figure 1:**
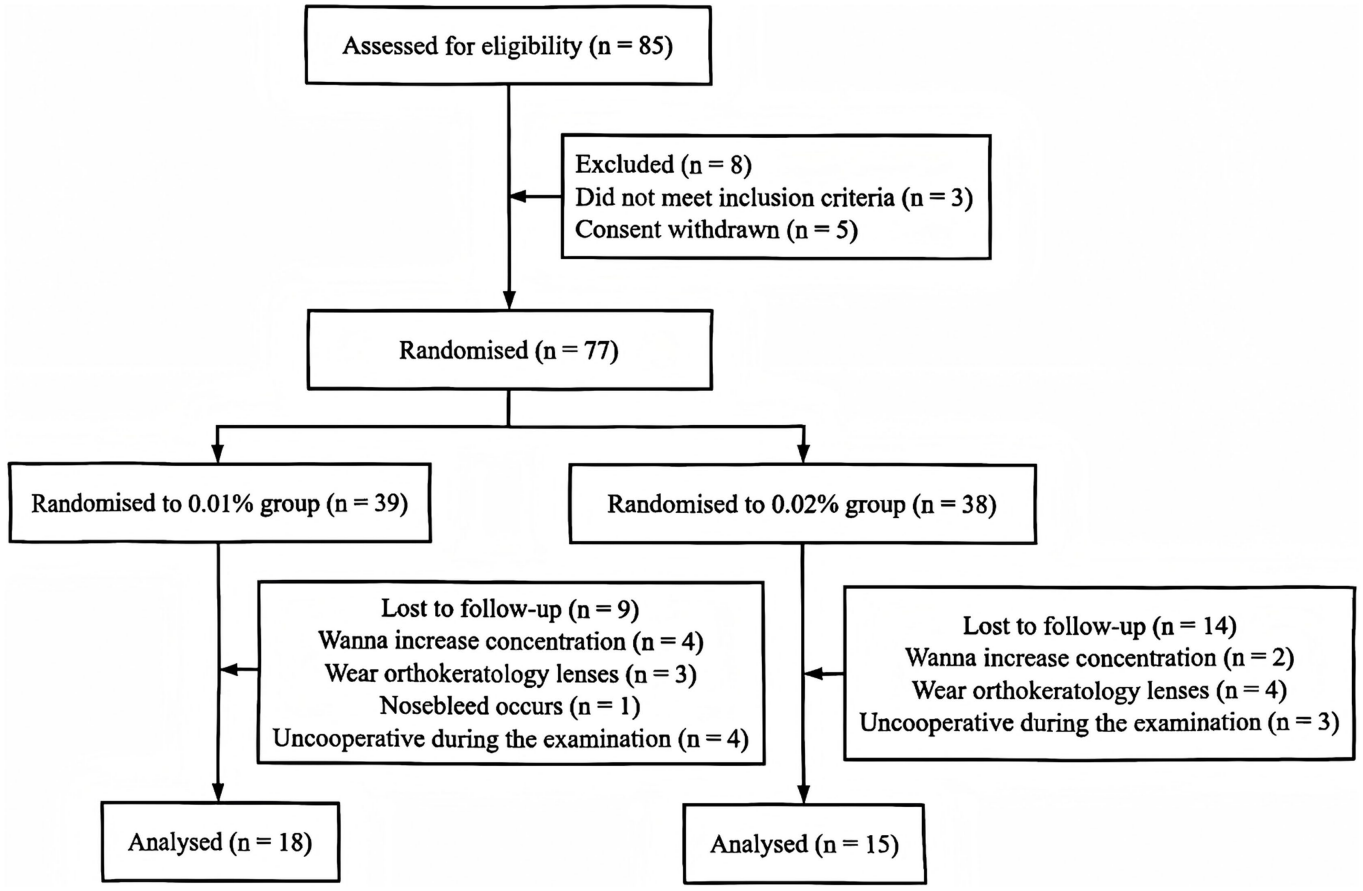
Flowchart of research progress.

This study adhered to the ethical guidelines outlined in the Declaration of Helsinki, ensuring informed consent, privacy protection, and the participants’ right to withdraw without consequences. It was conducted under the supervision of the Ethics Committee at the Second Affiliated Hospital of Anhui Medical University (Ethical approval number: YX2023-091) and registered with the China Clinical Trial Registry (ChiCTR2300077046). All patients and their guardians were fully informed of the study’s purpose and potential impacts, and written informed consent was obtained for using their medical records for data collection and analysis. This randomized controlled trial was conducted from January 2023 to December 2024 at the Ophthalmology Department of the Second Affiliated Hospital of Anhui Medical University, enrolling 85 children initially, with 18 children (36 eyes) in the 0.01% group and 15 children (30 eyes) in the 0.02% group after follow-up. There was a high dropout rate in the study, leading to a substantial difference between the initially selected sample and the final participant count. The details of the dropout situation are shown in Figure 1.

The inclusion criteria for patients were as follows: voluntary signing of informed consent, age between 6 and 10 years, normal intraocular pressure in both eyes, spherical refractive error ranging from −0.5 diopters (D) to −6.00D, cylindrical refractive error not exceeding 0.75D, best corrected visual acuity of 1.0 (LogMAR) or better, and the exclusive use of corrective spectacles for myopia. The exclusion criteria included systemic diseases related to ocular surface disorders, a history of ocular diseases of any etiology, previous ocular surgery, and a history of ocular medication use within the past 3 months.

### Experimental methods

Children with myopia who met the inclusion criteria and received treatment at the Ophthalmology Department of the Second Affiliated Hospital of Anhui Medical University were selected as study subjects. The children were randomly assigned to two groups: the 0.01% atropine group and the 0.02% atropine group. The 0.01% atropine group received one drop of 0.01% atropine ophthalmic solution in each eye at night, while the 0.02% atropine group received one drop of 0.02% atropine ophthalmic solution in each eye at night. Both groups underwent assessments of ocular surface disease index (OSDI), visual analogue scale (VAS), non-invasive ocular surface comprehensive analysis using the Keratograph 5M (OCULUS Optikgeräte GmbH, Wetzlar, Hesse, Germany), and meibomian gland automatic analysis using the Meibomian Gland Bio-image Analyzer V3 before treatment and at 1 month, 3 months, 6 months, and 12 months after treatment. All examinations were conducted under identical indoor conditions by an experienced examiner following a standardized procedure.

The Ocular Surface Disease Index (OSDI) was used to assess the severity of dry eye disease, covering three aspects: ocular symptoms, visual function, and environmental triggers. It consisted of twelve items, with a total score of 100 points. The higher the score, the more severe the dry eye symptoms. The Visual Analogue Scale (VAS) has been commonly used to assess the severity of pain. In this study, a seven-item ocular symptom scoring system was designed, including scores for burning sensation, itching, foreign body sensation, blurred vision, dryness, photophobia, and pain. Due to the younger average age of the patients and their relatively limited understanding compared to adults, we adopted a lower resolution VAS scale design. Each scale was 10 cm long, with no tick marks, only the endpoints labeled, and a resolution of 0–10, requiring patients to provide integer responses only. The total score was 70 points, with higher scores indicating more severe ocular symptoms.

The non-invasive ocular surface analyzer using the Keratograph 5M (OCULUS, Germany) was used to measure tear meniscus height (TMH), tear film break-up time, and to conduct meibomian gland imaging. The Keratograph 5M utilizes a penetrating technique to capture tear meniscus height under white light, without the need for fluorescein, which avoids stimulating tear secretion and does not interfere with the measurement of tear film break-up time. The device automatically acquires the first non-invasive tear film break-up time (fNIBUT) and the average non-invasive tear film break-up time (avNIBUT). The Keratograph 5M captured meibomian gland images using infrared transmission, and these images were subsequently imported into the Meibomian Gland Bio-image Analyzer V3 software for quantitative analysis.

The Meibomian Gland Bio-image Analyzer V3 is a quantitative analysis system that processes meibomian gland images using a corresponding algorithm to calculate meibomian gland biological parameters. Morphological parameters of the meibomian glands in the central five glands of the upper and lower eyelid conjunctivae were quantitatively analyzed by the same technician, with the final value being the average of the five glands. For repeated measurements of meibomian gland images from the same patient, consistency in the target glands was ensured. The average values of the meibomian gland biological parameters measured in the study included average gland diameter (avGD), average gland length (avGL), average gland area (avGA), deformation coefficient (DC), and gland visibility score (VS). We used the prefix “upper- (U-)” to denote upper meibomian gland parameters, and the prefix “lower- (L-)” to denote lower meibomian gland parameters.

### Statistical analysis

Data processing and statistical analysis for this study were conducted using SPSS 26.0 software. Chi-square tests were employed to compare the differences in gender data across various groups. Continuous variables were presented as mean ± standard deviation. The normality of continuous variable data was evaluated using the Kolmogorov–Smirnov test. For data such as age, axial length, and mean spherical equivalent that were not normally distributed, the Mann–Whitney U test was used to compare differences between groups. The Friedman test for multiple related samples was applied to assess intra-group differences in data at different time points before and after using eye drops in both the 0.01 and 0.02% groups. The Spearman test was employed to assess correlations between data, with correlation coefficients of *r* < 0.7 indicating low correlation, 0.7–0.9 indicating moderate correlation, and *r* ≥ 0.9 indicating high correlation. A significance level of *α* = 0.05 was set, with differences considered statistically significant when *p* < 0.05. The equations should be inserted in editable format from the equation editor.

## Results

### Patient characteristics

This study screened and enrolled 33 patients at the Ophthalmology Department of the Second Affiliated Hospital of Anhui Medical University from January 2023 to December 2024. The patients were randomly divided into the 0.01% group and the 0.02% group. The 0.01% group consisted of 18 patients (36 eyes), including 10 males (20 eyes) and 8 females (16 eyes). The 0.02% group consisted of 15 patients (30 eyes), including 8 males (16 eyes) and 7 females (14 eyes). No significant statistical differences were observed between the two groups in terms of gender, age, axial length, and spherical equivalent refractive error (all *p* > 0.05). The baseline characteristics of both groups are presented in Table 1.

**Table 1:** Basic information in two group.

Variable	0.01% Group	0.02% Group	*p*-value
Male/female (*n*)	10/8	8/7	0.898^*^
Age (years)	9.44 ± 1.65	8.67 ± 1.11	0.178^**^
Axial length (mm)	24.29 ± 1.09	23.99 ± 0.76	0.119^**^
Mean spherical equivalent (D)	−2.21 ± 1.55	−2.18 ± 1.54	0.781^**^

^*^Pearson’s χ^2^ test.

^**^Mann–Whitney U test.

### OSDI and VAS

In the 0.01% group, the comparison of OSDI questionnaire scores at different time points within the group showed no statistically significant differences (*χ*^2^ = 5.459, *p* = 0.243). In the 0.02% group, the comparison of OSDI questionnaire scores at different time points within the group showed statistically significant differences (*χ*^2^ = 9.929, *p* = 0.042). The OSDI score at 3 months of 0.02% atropine eye drop use was significantly higher compared to baseline (*p* = 0.006), and the score at 12 months of 0.02% atropine eye drop use was significantly lower compared to the 3-month score (*p* = 0.038).

The comparison of VAS scores at different time points within the 0.01% group showed no statistically significant differences (*χ*^2^ = 6.511, *p* = 0.164). In the 0.02% group, the comparison of VAS scores at different time points within the group showed statistically significant differences (*χ*^2^ = 11.830, *p* = 0.019). The VAS score at 3 months of 0.02% atropine eye drop use was significantly higher compared to baseline and 1 month (*p* = 0.009, *p* = 0.028). The VAS score at 6 months of 0.02% atropine eye drop use was significantly higher compared to baseline and 1 month (*p* = 0.015, *p* = 0.043) (Tables 2, 3).

**Table 2:** Changes in ocular surface parameters at different time points in the 0.01% group.

Variable	Baseline	1 month	3 months	6 months	12 months	*p*-value
OSDI score	3.47 ± 3.20	3.47 ± 2.37	6.13 ± 4.32	5.79 ± 4.88	4.63 ± 3.68	0.243^*^
VAS score	1.17 ± 1.25	2.11 ± 2.08	3.83 ± 3.28	2.22 ± 2.82	2.94 ± 3.75	0.164^*^
TMH (mm)	0.18 ± 0.06	0.18 ± 0.07	0.16 ± 0.04	0.18 ± 0.04	0.15 ± 0.04	0.652^*^
fNIBUT (s)	8.47 ± 5.37	9.35 ± 5.67	7.88 ± 4.82	9.34 ± 6.64	9.02 ± 5.32	0.074^*^
avNIBUT (s)	9.77 ± 5.46	12.07 ± 6.18	11.27 ± 5.01	12.22 ± 6.65	11.77 ± 5.53	0.233^*^
U-avGD (mm)	0.55 ± 0.17	0.63 ± 0.32	0.51 ± 0.15	0.47 ± 0.20	0.61 ± 0.39	0.139^*^
U-avGL (mm)	3.21 ± 0.49	3.55 ± 0.76	3.30 ± 0.59	3.19 ± 0.47	3.55 ± 0.80	0.466^*^
U-avGA (mm^2^)	1.29 ± 0.27	1.70 ± 1.00	1.30 ± 0.44	1.20 ± 0.43	1.68 ± 1.08	0.415^*^
U-DC	14.24 ± 9.15	14.46 ± 9.24	10.86 ± 5.71	10.27 ± 8.76	16.94 ± 14.42	0.525^*^
U-DV	5.25 ± 1.33	4.86 ± 0.91	5.19 ± 1.29	4.47 ± 1.17	4.38 ± 0.79	0.274^*^
L-avGD (mm)	0.70 ± 0.27	0.68 ± 0.35	0.69 ± 0.39	0.67 ± 0.28	0.56 ± 0.18	0.426^*^
L-avGL (mm)	1.93 ± 0.46	1.75 ± 0.31	1.71 ± 0.48	1.73 ± 0.49	1.76 ± 0.40	0.917^*^
L-avGA (mm^2^)	0.92 ± 0.35	0.81 ± 0.25	0.78 ± 0.33	0.77 ± 0.23	0.77 ± 0.32	0.994^*^
L-DC	20.83 ± 10.36	25.48 ± 30.03	33.88 ± 48.95	28.48 ± 28.24	15.02 ± 9.49	0.463^*^
L-DV	6.73 ± 1.89	6.71 ± 1.58	6.41 ± 1.76	7.01 ± 2.15	6.77 ± 2.58	0.308^*^

^*^Friedman test.

**Table 3:** Changes in ocular surface parameters at different time points in the 0.02% group.

Variable	Baseline	1 month	3 months	6 months	12 months	*p*-value
OSDI score	6.40 ± 5.20	9.08 ± 7.60	10.72 ± 4.31^a^	9.23 ± 4.60	6.99 ± 5.08^c^	0.042^*^
VAS score	2.14 ± 2.45	2.79 ± 3.87	7.86 ± 7.26^a^^,^^b^	6.79 ± 5.85^a^^,^^b^	4.79 ± 3.79	0.019^*^
TMH (mm)	0.21 ± 0.07	0.16 ± 0.05	0.16 ± 0.06	0.18 ± 0.05	0.15 ± 0.05^a^^,^^b^^,^^c^	0.041^*^
fNIBUT (s)	9.49 ± 6.62	7.35 ± 4.96	9.48 ± 6.31	9.62 ± 5.63	11.15 ± 7.44	0.266^*^
avNIBUT (s)	11.82 ± 6.69	8.96 ± 5.39	10.98 ± 5.85	11.23 ± 5.66	12.82 ± 6.38	0.122^*^
U-avGD (mm)	0.45 ± 0.08	0.39 ± 0.08	0.39 ± 0.06	0.48 ± 0.08	0.46 ± 0.11	0.141^*^
U-avGL (mm)	3.23 ± 0.51	3.30 ± 0.83	3.33 ± 0.54	3.25 ± 0.72	3.38 ± 0.62	0.525^*^
U-avGA (mm^2^)	1.24 ± 0.27	1.14 ± 0.46	1.11 ± 0.25	1.22 ± 0.33	1.30 ± 0.42	0.262^*^
U-DC	9.00 ± 2.88	7.68 ± 2.64	7.38 ± 3.16	11.87 ± 4.99	10.39 ± 5.45	0.133^*^
U-DV	4.66 ± 1.32	5.80 ± 1.94	4.44 ± 1.74^b^	5.65 ± 1.68^c^	4.98 ± 1.07^c^	0.027^*^
L-avGD (mm)	0.60 ± 0.26	0.51 ± 0.11	0.55 ± 0.08	0.59 ± 0.10	0.53 ± 0.08	0.704^*^
L-avGL (mm)	1.81 ± 0.69	2.24 ± 0.30^a^	2.12 ± 0.44	2.13 ± 0.55^b^	2.01 ± 0.33	0.017^*^
L-avGA (mm^2^)	0.78 ± 0.36	0.93 ± 0.28	0.97 ± 0.30	0.96 ± 0.27	0.90 ± 0.20	0.704^*^
L-DC	12.58 ± 5.23	11.76 ± 4.50	16.45 ± 9.48	16.72 ± 10.52	11.02 ± 2.39	0.289^*^
L-DV	5.08 ± 1.42	5.57 ± 1.54	6.25 ± 1.42	6.98 ± 1.61^a^	5.68 ± 1.47^a^	0.044^*^

^*^Friedman test.

a Compared with baseline, *p* < 0.05.

b Compared with 1 month, *p* < 0.05.

c Compared with 3 months, *p* < 0.05.

### TMH and NIBUT

The comparison of TMH at different time points within the 0.01% group showed no statistically significant differences (*χ*^2^ = 2.459, *p* = 0.652). In the 0.02% group, the comparison of TMH at different time points within the group showed statistically significant differences (*χ*^2^ = 9.983, *p* = 0.041). TMH at 12 months of 0.02% atropine eye drop use was significantly lower compared to baseline, 1 month, and 3 months (*p* = 0.005, *p* = 0.022, *p* = 0.018).

The comparison of fNIBUT at different time points within both the 0.01% group and the 0.02% group showed no statistically significant differences (*χ*^2^ = 8.525, *p* = 0.074; *χ*^2^ = 5.217, *p* = 0.266). Similarly, the comparison of avNIBUT at different time points within both the 0.01% group and the 0.02% group also showed no statistically significant differences (*χ*^2^ = 5.578, *p* = 0.233; *χ*^2^ = 7.278, *p* = 0.122) (Tables 2, 3).

### Meibomian gland data

The comparison of U-avGD at different time points within the 0.01% group and the 0.02% group showed no statistically significant differences (*χ*^2^ = 6.937, *p* = 0.139; *χ*^2^ = 6.908, *p* = 0.141). The comparison of U-avGL at different time points within both the 0.01 and 0.02% groups also showed no statistically significant differences (*χ*^2^ = 3.577, *p* = 0.466; *χ*^2^ = 3.200, *p* = 0.525). The comparison of U-avGA at different time points within both the 0.01 and 0.02% groups revealed no statistically significant differences (*χ*^2^ = 3.933, *p* = 0.415; *χ*^2^ = 5.255, *p* = 0.262). There were no statistically significant differences in the comparison of U-DC at different time points within both the 0.01 and 0.02% groups (*χ*^2^ = 3.200, *p* = 0.525; *χ*^2^ = 7.062, *p* = 0.133). The comparison of U-VS within the 0.01% group showed no statistical significance (*χ*^2^ = 5.133, *p* = 0.274), while in the 0.02% group, significant differences were found (*χ*^2^ = 10.987, *p* = 0.027). The U-VS at 3 months of 0.02% atropine eye drop use was significantly lower compared to 1 month (*p* = 0.006), and at 6 months, it was significantly higher compared to 3 months (*p* = 0.006). At 12 months, U-VS was significantly higher compared to 3 months (*p* = 0.034).

The comparison of L-avGD at different time points within the 0.01 and 0.02% groups showed no statistically significant differences (*χ*^2^ = 3.853, *p* = 0.426; *χ*^2^ = 2.175, *p* = 0.704). The comparison of L-avGL within the 0.01% group showed no significant differences (*χ*^2^ = 0.954, *p* = 0.917). However, in the 0.02% group, the comparison of L-avGL at different time points showed statistically significant differences (*χ*^2^ = 12.013, *p* = 0.017). L-avGL was significantly higher at 1 month of 0.02% atropine eye drop use compared to baseline (*p* = 0.001), and at 6 months, it was significantly lower compared to 1 month (*p* = 0.013). The comparison of L-avGA at different time points within both the 0.01 and 0.02% groups showed no statistically significant differences (*χ*^2^ = 0.230, *p* = 0.994; *χ*^2^ = 2.174, *p* = 0.704). The comparison of L-DC at different time points within both the 0.01 and 0.02% groups showed no statistically significant differences (*χ*^2^ = 3.600, *p* = 0.463; *χ*^2^ = 4.987, *p* = 0.289). The comparison of L-VS within the 0.01% group showed no statistical significance (*χ*^2^ = 4.800, *p* = 0.308), whereas in the 0.02% group, the comparison of L-VS showed significant differences (*χ*^2^ = 9.787, *p* = 0.044). L-VS was significantly higher at 6 months and 12 months of 0.02% atropine eye drop use compared to baseline (*p* = 0.003, *p* = 0.034) (Tables 2, 3).

### Correlations between U-VS and L-VS with OSDI, VAS, and TMH in the 0.02% group at 3, 6, and 12 months of medication use

At 3 months of medication use, a positive correlation was found between the change in U-VS and the change in VAS (*r* = 0.542, *p* = 0.037), but there was no correlation between the change in U-VS and the change in OSDI or TMH (*r* = 0.07, *p* = 0.979; *r* = −0.168, *p* = 0.375). The change in L-VS showed a positive correlation with the change in VAS (*r* = 0.614, *p* = 0.015), but there was no correlation between the change in L-VS and the change in OSDI or TMH (*r* = −0.084, *p* = 0.765; *r* = 0.041, *p* = 0.828) (Table 4).

**Table 4:** Correlation analysis between U-DV and L-DV with OSDI, VAS, and TMH in the 0.02% group at 3 months of treatment.

Variable	U-DV		L-DV	
	*r*-value	*p*-value	*r*-value	*p*-value
OSDI score	0.07	0.979	−0.084	0.765
VAS score	0.542	0.037^*^	0.614	0.015^*^
TMH (mm)	−0.168	0.375	0.041	0.828

^*^There is a low correlation between the two.

At 6 months of medication use, no correlation was found between the change in U-VS and the change in OSDI, VAS, or TMH (*r* = 0.238, *p* = 0.394; *r* = 0.233, *p* = 0.402; *r* = 0.130, *p* = 0.494). The change in L-VS showed a positive correlation with the change in OSDI (*r* = 0.610, *p* = 0.016), but there was no correlation between the change in L-VS and the change in VAS or TMH (*r* = 0.278, *p* = 0.315; *r* = 0.271, *p* = 0.148) (Table 5).

**Table 5:** Correlation analysis between U-DV and L-DV with OSDI, VAS, and TMH in the 0.02% group at 6 months of treatment.

Variable	U-DV		L-DV	
	*r*-value	*p*-value	*r*-value	*p*-value
OSDI score	0.238	0.394	0.610	0.016^*^
VAS score	0.233	0.402	0.278	0.315
TMH (mm)	0.130	0.494	0.271	0.148

^*^There is a low correlation between the two.

At 12 months of medication use, a positive correlation was found between the change in U-VS and the change in TMH (*r* = 0.521, *p* = 0.003), however no correlation was found between the change in U-VS and the changes in OSDI or VAS (*r* = −0.292, *p* = 0.290; *r* = 0.138, *p* = 0.623). The change in L-VS showed no correlation with the change in OSDI, VAS, or TMH (*r* = 0.361, *p* = 0.186; *r* = 0.291, *p* = 0.292; *r* = −0.133, *p* = 0.485) (Table 6).

**Table 6:** Correlation analysis between U-DV and L-DV with OSDI, VAS, and TMH in the 0.02% group at 12 months of treatment.

Variable	U-DV		L-DV	
	*r*-value	*p*-value	*r*-value	*p*-value
OSDI score	−0.292	0.290	0.361	0.186
VAS score	0.138	0.623	0.291	0.292
TMH (mm)	0.521	0.003^*^	−0.133	0.485

^*^There is a low correlation between the two.

## Discussion

Myopia is a common refractive error of the eye, often marked by blurred vision when viewing distant objects. The underlying cause is an elongated axial length of the eyeball or an excessively strong refractive power of the cornea and lens or both, which results in parallel light rays focusing in front of the retina instead of directly on it. Myopic maculopathy and optic nerve damage associated with high myopia are common causes of irreversible vision loss in East Asia (14–18). The primary risk factor for the development of complications related to pathological myopia is the elongation of the eye’s axial length (19–21). As a result, controlling myopia progression and slowing the elongation of axial length have become key objectives in the prevention and management of myopia. Multiple clinical studies have shown that low-concentration atropine eye drops can effectively delay the progression of myopia and have been widely applied (22–24).

The Ocular Surface Disease Index (OSDI) is an instrument designed to evaluate symptoms of dry eye and their impact on quality of life. Due to its cost-effectiveness and simplicity, it has been widely adopted. The Visual Analogue Scale (VAS) is a simple and effective subjective symptom assessment tool that converts subjective symptoms into quantifiable values, making it easier for doctors to analyze and make judgments. During the one-year trial observation, there were no significant changes in the subjective OSDI questionnaire and VAS scale scores in the 0.01% group, which aligns with the findings of Cheng J et al. (25). A 2-week study found that adults using 0.05% atropine eye drops were more likely to experience dry eye compared to those using 0.01%. The 0.05% concentration significantly affected the tear film, while the 0.01% concentration had little effect (26). Our findings also indicate that 0.01% atropine has no significant impact on the ocular surface. However, in the 0.02% group, the OSDI scores significantly increased at 3 months of using the eye drops compared to baseline, and significantly decreased at 12 months when compared to 3 months. The OSDI score decreased at 12 months, likely because although the tear meniscus height significantly decreased at 12 months, the image values of both the upper and lower meibomian glands significantly increased compared to previous measurements. The active secretion of lipids alleviated ocular discomfort. The VAS scores in the 0.02% group were significantly higher at 6 months and 3 months compared to 1 month and baseline.

These findings suggested that 0.02% atropine eye drops may cause more pronounced ocular discomfort compared to 0.01% atropine eye drops. This discomfort typically began to manifest after about three months of use, presenting as symptoms such as dry eyes, burning sensations, and blurred vision. As the treatment period progressed, the patients’ ocular discomfort gradually lessened, indicating an adaptive improvement. This process may be related to the eye’s gradual adaptation to the drug and the diminishing side effects over time. After initially experiencing discomfort, patients often became more accustomed to the medication, eventually reaching a more stable state.

Tear meniscus height (TMH) refers to the vertical height at the center of the crescent-shaped liquid meniscus formed between the eyelid margin and the lower eyelid. The first noninvasive tear film breakup time (fNIBUT) and average noninvasive tear film breakup time (avNIBUT) measured by non-invasive ocular surface analyzers offer advantages over traditional slit-lamp fluorescence staining measurements, such as greater comfort, objectivity, and convenience. Tear fluid layer that covers the surface of the eye isprimarily composed of the lipid layer, aqueous layer, and mucin layer. Each layer plays a crucial role in preserving the stability and function of the tear film, working together to ensure ocular health. In the 0.01 and 0.02% groups, there were no significant changes in fNIBUT and avNTBUT throughout the one-year period of eye drop use. However, in the 0.01% group, there were no significant changes in TMH during the year of eye drop use. In contrast, the 0.02% group showed a notable decrease in TMH at 12 months compared to baseline, 1 month, and 3 months of use.

This suggested that the 0.01% concentration of atropine eye drops has a negligible impact on tear meniscus height and tear film breakup time. Sánchez-Ríos A et al. (27) studied the effects of 1% atropine on the eyes of New Zealand rabbits for one month. They found a decrease in both tear secretion and tear film breakup time, with mild inflammation observed in the lacrimal gland tissue upon histopathological examination. This study provides experimental evidence for the risk of dry eye disease. The reduction in TMH observed in the 0.02% group at 12 months may be related to seasonal factors, as most patients were followed up during winter when the climate is drier. Additionally, the heating system in Hefei further dried the indoor air, which may have exacerbated the sensation of dry eyes. Another possible reason was the 12-month follow-up period, during which some patients advanced to the next grade level. This could have increased academic pressure, with more homework and extended near-vision use, contributing to the decline in tear meniscus height.

The meibomian gland automatic analysis software quantifies the morphology, function, and loss of meibomian glands, providing more precise biological parameters for clinical use. This software had shown good applicability in a clinical study evaluating the functional and morphological changes of meibomian glands in cataract surgery patients (28). The average gland length (avGL) and average gland area (avGA) of the central glands reflect the functional condition of the glands. The average gland diameter (avGD) can partially reflect the degree of gland obstruction. The deformation coefficient (DC) shows the degree of variation in gland morphology. A lower DC indicates that the gland’s morphology is closer to normal, while a higher DC indicates more abnormal morphology. The gland visibility score (VS) reflects the content of the glandular acini, which continuously secrete a mixture of oils and proteins. Therefore, a higher VS suggests a higher lipid content in the gland, while a lower VS indicates lower lipid content.

In the 0.01% group, no significant changes were observed in the quantification data of the upper and lower meibomian glands over the one-year observation period with 0.01% atropine eye drops. In the 0.02% group, during the one-year observation period with 0.02% atropine eye drops, no significant changes were found in U-avGD, U-avGL, U-avGA, or U-DC of the upper meibomian gland. However, U-VS of the upper meibomian glands in the 0.02% group decreased significantly at 3 months of eye drop use compared to 1 month. At both 6 and 12 months, U-VS significantly increased compared to 3 months. The changes in U-VS suggested that 0.02% atropine eye drops may suppress lipid secretion in the meibomian glands, with the peak suppression occurring around 3 months, followed by gradual weakening and eventual disappearance of this effect. For the lower meibomian glands in the 0.02% group, there were no significant changes in L-avGD, L-avGA, or L-DC throughout the one-year observation period. However, L-avGL of the lower meibomian glands at 1 month of eye drop use was significantly higher compared to baseline, and at 6 months, it significantly decreased compared to 1 month. This suggested that the length of the lower meibomian glands may undergo a compensatory increase at 1 month of treatment. Additionally, L-VS of the lower meibomian glands at both 6 and 12 months was significantly higher than the baseline values, indicating a good lipid content in the glands. The increased lipid secretion in the glands was considered to be a rebound effect in the meibomian glands following stimulation. Based on the analysis of the L-VS and L-avGL in the lower meibomian glands, it was hypothesized that there was a compensatory mechanism in the meibomian glands, with a significant compensatory ability in the lower meibomian glands.

A correlation analysis was performed to investigate the changes in U-VS and L-VS in the 0.02% group in relation to the OSDI, VAS, and TMH. It was found that at 3 months of medication use, the changes in VAS showed a low correlation with both U-VS and L-VS. At 6 months, the changes in L-VS were lowly correlated with the OSDI. At 12 months, a low correlation was observed between the changes in U-VS and TMH. The higher VS, the more it indicates lipid blockage within the meibomian glands, leading to compensatory tear secretion increase, which in turn intensifies the patient’s subjective discomfort. The patients’ subjective sensation of ocular discomfort was related to the meibomian gland density values and the lipid secretion within the glands.

This study utilized an artificial intelligence-based meibomian gland automatic analysis software, which captured high-resolution infrared meibomian gland images through a standardized imaging process. It employed digital morphological parameters to construct a multidimensional quantification assessment system, systematically analyzing the correlation between microscopic structural changes and functional status of the meibomian glands before and after intervention.

However, there are some limitations in this research. The average age of the two groups of patients was relatively young, which may lead to ambiguous expression of subjective feelings. This study primarily focused on the 0.01 and 0.02% concentrations, which exhibited a relatively small difference. Future research will compare the 0.01% concentration with the 0.05% concentration in order to identify potentially more valuable clinical indicators and their trends. Additionally, the sample size for both groups was small, which could reduce the ability to detect small effect sizes and increase the risk of type II errors. The study lacked a placebo control group. Moreover, the follow-up period was short. Future research could increase the sample size, lengthen the follow-up period, and explore the possibility of meibomian gland morphological and functional recovery after discontinuing low-concentration atropine eye drops. Moving forward, We will place greater emphasis on the effects of low-concentration atropine eye drops on ocular surface health and further explore alterations in meibomian gland function and structure.

In summary, based on the subjective symptom scores and objective meibomian gland quantification data from the 0.02% group, 0.02% atropine eye drops had a negligible impact on tear film breakup time, primarily affecting the lipid secretion within the meibomian glands and tear meniscus height, thus increasing the patients’ subjective discomfort, especially in the upper meibomian glands. This suggestes a complex compensatory mechanism within the meibomian glands. Clinically, patients using 0.02% atropine eye drops for a prolonged period should have their ocular surface health monitored. On the other hand, 0.01% atropine eye drops did not affect ocular surface health, meibomian glands, or subjective symptoms, indicating good safety. Myopia may be a potential risk factor for dry eye disease (29, 30). Currently, low-concentration atropine is often used alongside other corrective methods to help slow the progression of myopia. The use of low-concentration atropine in combination with orthokeratology lenses is an effective approach for managing myopia progression, but orthokeratology lenses may exert mechanical pressure on the meibomian glands, potentially affecting their normal lipid secretion function. Long-term use of orthokeratology lenses may bring the lens in direct contact with the corneal surface, altering the structural and pressure distribution around the meibomian glands, thus inhibiting their secretory activity. Additionally, the effect of orthokeratology lenses on the ocular surface microenvironment should not be underestimated. Wearing lenses can change the distribution and flow of tear fluid, affecting its stability and further compromising the stability of the tear film. Since the flow and distribution of tears are closely related to lipid discharge from the meibomian glands, any disruption in tear distribution may prevent the effective expulsion of lipids from the glands, leading to lipid accumulation or inefficient excretion. The interaction of these factors may lead to increased ocular discomfort and even raise the risk of developing dry eye disease.

Therefore, when administering low-concentration atropine eye drops as a clinical intervention for myopia progression and axial elongation, it is crucial to simultaneously monitor ocular surface health, especially when combined with orthokeratology lenses. A thorough assessment and monitoring of ocular surface function should be prioritized to optimize treatment outcomes and minimize the potential risk of complications.

## Conclusion

0.01% atropine eye drops had no significant effect on the ocular surface, meibomian glands, or subjective symptoms. 0.02% atropine eye drops affected the lipid secretion of meibomian glands, tear meniscus height and subjective discomfort. 0.02% atropine eye drops affected the lipid secretion of upper meibomian glands. Subjective ocular sensations were associated with the meibomian gland develop value and lipid secretion within the glands. It is recommended to focus on ocular surface health when using low-concentration atropine eye drops for myopia management.

## Data Availability

The raw data supporting the conclusions of this article will be made available by the authors, without undue reservation.
